# Salicylic Acid: A Double-Edged Sword for Programed Cell Death in Plants

**DOI:** 10.3389/fpls.2018.01133

**Published:** 2018-08-07

**Authors:** Ana Radojičić, Xin Li, Yuelin Zhang

**Affiliations:** ^1^Department of Botany, The University of British Columbia, Vancouver, BC, Canada; ^2^The Michael Smith Laboratories, The University of British Columbia, Vancouver, BC, Canada

**Keywords:** salicylic acid, hypersensitive reaction, programed cell death, effector-triggered immunity, plant immunity

## Abstract

In plants, salicylic acid (SA) plays important roles in regulating immunity and programed cell death. Early studies revealed that increased SA accumulation is associated with the onset of hypersensitive reaction during resistance gene-mediated defense responses. SA was also found to accumulate to high levels in lesion-mimic mutants and in some cases the accumulation of SA is required for the spontaneous cell death phenotype. Meanwhile, high levels of SA have been shown to negatively regulate plant cell death during effector-triggered immunity, suggesting that SA has dual functions in cell death control. The molecular mechanisms of how SA regulates cell death in plants are discussed.

Salicylic acid (SA) is a plant hormone that plays key roles in defense signaling (Vlot et al., 2009). Pathogen infection induces SA biosynthesis and accumulation. Two groups of Arabidopsis mutants, *salicylic acid induction deficient2* (*sid2*) and *enhanced disease susceptibility5* (*eds5*), are deficient in pathogen-induced SA accumulation and exhibit increased susceptibility to biotrophic pathogens (Nawrath and Metraux, 1999; Dewdney et al., 2000). *sid2* mutants carry mutations in the isochorismate synthase ICS1, suggesting that SA is synthesized from chorismate following pathogen infection via ICS1 (Wildermuth et al., 2001). *EDS5* encodes a multi-antimicrobial extrusion protein (MATE) transporter (Nawrath et al., 2002). The exact role of EDS5 in SA metabolism is unclear. It is likely to be involved in exporting SA or a precursor of SA out of plastids (Serrano et al., 2013).

SA is perceived by two groups of receptors, NONEXPRESSOR OF PATHOGENESIS-RELATED GENES1 (NPR1) and NPR3/NPR4, all of which display high affinity with SA (Fu et al., 2012; Wu et al., 2012; Manohar et al., 2015; Ding et al., 2018). However, they have opposite roles in transcriptional regulation of defense gene expression (Ding et al., 2018). NPR1 functions as a transcriptional activator that promotes SA-induced defense gene expression and pathogen resistance (Fan and Dong, 2002). Loss of NPR1 results in reduced SA-induced *PR* gene expression and increased susceptibility to pathogens (Cao et al., 1994; Delaney et al., 1995). On the other hand, NPR3 and NPR4 serve as redundant transcriptional co-repressors that prevent activation of defense gene expression when the SA level is low (Ding et al., 2018). When SA levels are high, SA inhibits the transcriptional repression activity of NPR3/NPR4 to activate the expression of SA-responsive genes. The NPR4-4D mutant protein that is unable to bind SA constitutively represses defense gene expression and blocks SA-induced immunity, rendering the mutant plants with enhanced disease susceptibility (Ding et al., 2018). Regulation of defense genes by NPR1 and NPR3/NPR4 is directly facilitated by a group of redundant bZIP transcription factors, including TGA2, TGA5, and TGA6, which interact with both NPR1 and NPR3/NPR4 (Zhang et al., 1999, 2003, 2006; Despres et al., 2000; Zhou et al., 2000).

Increased SA accumulation is associated with hypersensitive response (HR), a form of programed cell death often induced by effector-triggered immunity (ETI), as well as spontaneous cell death in lesion-mimic mutants. Early studies showed that activation of *N* gene-mediated defense responses by tobacco mosaic virus led to about 20-fold increase in endogenous SA levels in the infected tobacco leaves (Malamy et al., 1990). Activation of ETI by *Pseudomonas* effectors AvrRpm1 and AvrRpt2 in Arabidopsis also results in dramatic increases in local SA levels in a SID2 and EDS5-dependent manner (Nawrath and Metraux, 1999). Meanwhile, in mutants with spontaneous cell death, SA accumulates at much higher levels than in wild type (Bruggeman et al., 2015). However, in autoimmune mutants with no spontaneous lesion formation, such as *suppressor of npr1-1, constitutive1* (*snc1*) and *defense, no death1* (*dnd1*), SA levels are still dramatically increased (Yu et al., 1998; Li et al., 2001), suggesting that cell death is not required for the activation of SA biosynthesis and high levels of SA alone are not sufficient to activate cell death.

Salicylic acid has been shown to be required for spontaneous cell death in several lesion-mimic mutants (**Table 1**). Treatment with low levels of SA activates runaway cell death in *lesion simulating disease 1* (*lsd1*) (Dietrich et al., 1994). Blocking SA accumulation by expressing the SA hydroxylase encoded by the bacterial *NahG* gene suppresses lesion formation in *lsd6*, *lsd7*, *accelerated cell death 6* (*acd6*), and *acd11* mutants (Weymann et al., 1995; Rate et al., 1999; Brodersen et al., 2005). In the *syntaxin of plants 121* (*syp121*) *syp122* double mutant, spontaneous cell death is also attenuated when SA biosynthesis or SA perception is blocked (Zhang et al., 2007). However, not all lesion-mimic mutants require SA accumulation for activation of spontaneous cell death. For example, expression of *NahG* does not affect lesion formation in *lsd2* and *lsd4* mutants (Dietrich et al., 1994; Hunt et al., 1997).

**Table 1:** SA levels and cell death phenotypes of *Arabidopsis thaliana* mutants.

Mutant	SA levels	Cell death phenotype	Reference
*lsd1*	High	Spontaneous cell death	Dietrich et al., 1994
*lsd2*	ND^∗^	Spontaneous cell death	Dietrich et al., 1994
*lsd2 nahG*	Low	Spontaneous cell death	Dietrich et al., 1994; Hunt et al., 1997
*lsd4*	ND^∗^	Spontaneous cell death	Dietrich et al., 1994
*lsd4 nahG*	Low	Spontaneous cell death	Dietrich et al., 1994; Hunt et al., 1997
*lsd6*	High	Spontaneous cell death	Weymann et al., 1995
*lsd6 nahG*	Low	No spontaneous cell death	Weymann et al., 1995
*lsd7*	High	Spontaneous cell death	Weymann et al., 1995
*lsd7 nahG*	Low	No spontaneous cell death	Weymann et al., 1995
*acd6*	High	Spontaneous cell death	Rate et al., 1999
*acd6 nahG*	Low	No spontaneous cell death	Rate et al., 1999
*acd11*	High	Spontaneous cell death	Brodersen et al., 2005
*acd11 nahG*	Low	No spontaneous cell death	Brodersen et al., 2005
*syp121 syp122*	High	Spontaneous cell death	Zhang et al., 2007
*syp121 syp122 nahG*	Low	Reduced spontaneous cell death	Zhang et al., 2007
*syp121 syp122 sid2*	Low	Reduced spontaneous cell death	Zhang et al., 2007
*snc1*	High	No spontaneous cell death	Li et al., 2001
*dnd1*	High	No spontaneous cell death; reduced AvrRpt2-induced cell death	Yu et al., 1998
*dnd2*	High	No spontaneous cell death; reduced AvrRpt2-induced cell death	Jurkowski et al., 2004
*agd2*	High	Spontaneous cell death; reduced AvrRpt2- and AvrRpm1-induced cell death	Rate and Greenberg, 2001
*agd2 nahG*	Low	Spontaneous cell death; restored AvrRpm1-induced cell death	Rate and Greenberg, 2001
*agd2 npr1*	ND^∗^	Reduced spontaneous cell death; restored AvrRpt2-induced and AvrRpm1-induced cell death	Rate and Greenberg, 2001
*hrl1*	High	Spontaneous cell death; reduced AvrRpm1-induced cell death	Devadas and Raina, 2002
*hrl1 nahG*	Low	Delayed spontaneous cell death; restored AvrRpm1-induced cell death	Devadas and Raina, 2002
*hrl1 npr1*	High	Delayed spontaneous cell death; restored AvrRpm1-induced cell death	Devadas and Raina, 2002
*npr3 npr4*	WT-like	No spontaneous cell death; reduced AvrRpt2-induced cell death	Zhang et al., 2006; Fu et al., 2012


^∗^ND, not determined; WT, wild type.

Interestingly, pre-treatment of Arabidopsis Col-0 plants with SA blocks HR activated by *Pseudomonas syringae* pv *maculicola* (*P.s.m.*) ES4326 carrying *avrRpm1* (Devadas and Raina, 2002). In transgenic plants overexpressing NPR1, activation of cell death by the bacteria is also attenuated (Rate and Greenberg, 2001). In addition, increased ion leakage was observed in *eds5-3* compared to wild type following treatment with *Pseudomonas syringae* pv *tomato* (*P.s.t.*) DC3000 with *avrRpt2* (**Figure 1A**), indicating that AvrRpt2-induced cell death is enhanced in *eds5-3*. These findings suggest that activation of SA signaling plays an important role in negative regulation of cell death during ETI.

**FIGURE 1:**
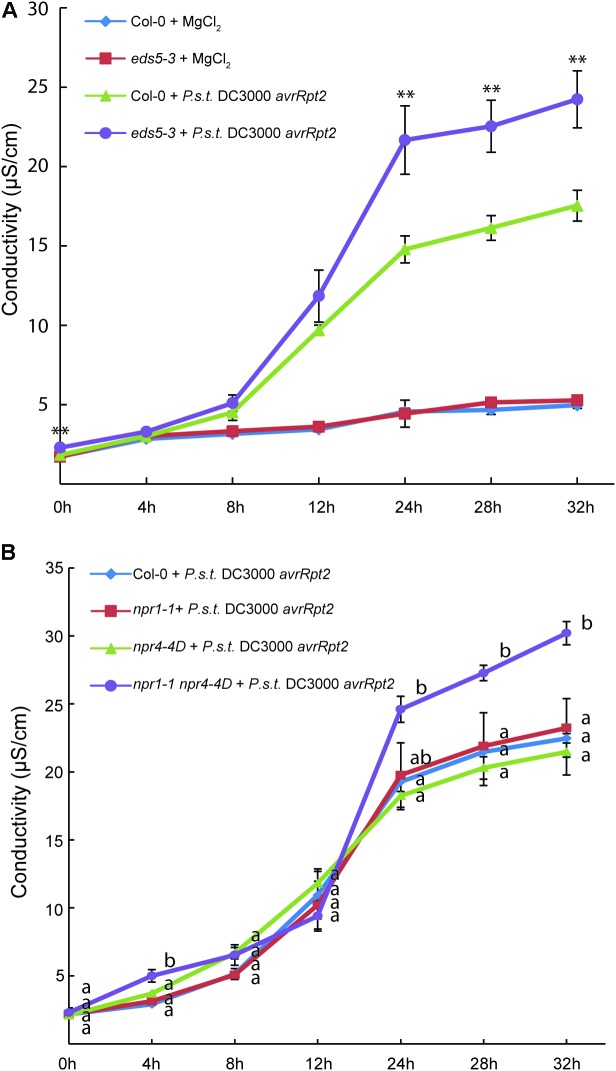
Analysis of ion leakage in *eds5-3*, *npr1-1*, *npr4-4D*, and *npr1-1 npr4-4D* plants after treatment with *P.s.t.* DC3000 *avrRpt2*. Leaves of 4-week-old plants of the indicated genotypes grown under 12 h/12 h light/dark photoperiod at 23°C were infiltrated with mock (10 mM MgCl_2_) or *P.s.t.* DC3000 *avrRpt2* (OD_600_ = 0.02). For each plant, two leaves were infiltrated and one leaf disk was cut from each leaf immediately after infiltration. The leaf disks were subsequently washed twice in distilled water. Six leaf disks from three plants, representing one biological replicate, were transferred into a 50-ml plastic tube containing 20 ml of distilled water and electrical conductivity was measured at different time points after infiltration using a VWR EC meter (Model 2052). Each data point on the graph represents the mean ± SD of three biological replicates. In **(A)**, Two-tailed *t*-test was performed for each time point between wild type (Col-0) and *eds5-3* plants treated with *P.s.t.* DC3000 *avrRpt2* (^∗∗^*p* < 0.01). In **(B)**, one way ANOVA with *post hoc* Tukey HSD test was performed for each time point among the different genotypes. Different letters (a,b) indicate statistically significant differences between the samples (*p* < 0.01).

Consistent with the role of pathogen-induced SA in negative regulation of cell death in ETI, enhanced cell death was observed in the *npr1-1* mutant compared to wild type following treatment with *P.s.m.* ES4326 carrying *avrRpm1* (Rate and Greenberg, 2001), suggesting that perception of SA by NPR1 is critical for the attenuation of AvrRpm1-induced cell death. When *npr1-1*, *npr4-4D*, and the *npr1-1 npr4-4D* double mutant plants were challenged with *P.s.t.* DC3000 carrying *avrRpt2*, cell death in the *npr1-1* and *npr4-4D* single mutants was similar to that in wild type, whereas *npr1-1 npr4-4D* exhibited enhanced cell death (**Figure 1B**), suggesting that *npr1-1* and *npr4-4D* have additive effect on AvrRpt2-induced cell death. These data also suggest that SA signaling mediated by both NPR1 and NPR3/NPR4 plays critical roles in dampening cell death during ETI.

Consistent with the effects of pathogen-induced SA accumulation on inhibition of HR, avirulent pathogen-induced cell death in several autoimmune mutants with high SA levels was found to be greatly reduced. For example, cell death induced by *P.s.m.* ES4326 strains carrying *avrRpt2* or *avrRpm1* is dramatically reduced in *aberrant growth and death2* (*agd2*) plants (Rate and Greenberg, 2001). The reduced cell death can be restored back to wild type level by introducing *NahG* or *npr1-1* into *agd2*, suggesting that the high SA level in *agd2* is responsible for the suppression of cell death activated during ETI. In the *hypersensitive response like lesions1* (*hrl1*) mutant, cell death induced by AvrRpt2 and AvrRpm1 is also greatly reduced (Devadas and Raina, 2002). Similarly, introducing *NahG* or *npr1-1* into *hrl1* leads to restoration of RPM1-mediated cell death. In another class of autoimmune mutants, including *dnd1* and *dnd2*, gene-for-gene resistance is normal, but there is almost no HR following infection by avirulent bacterial pathogens (Yu et al., 1998; Jurkowski et al., 2004). Both *dnd1* and *dnd2* accumulate high levels of SA in the absence of pathogen infection, which is likely responsible for the lack of ETI-induced HR in these mutants.

Arabidopsis NPR3 and NPR4 function redundantly in negative regulation of defense gene expression. *npr3 npr4* double mutants accumulate similar levels of SA as wild type plants, but constitutively express *PR* genes and exhibit enhanced resistance to virulent pathogens (Zhang et al., 2006). Interestingly, HR activated by AvrRpt2 is almost completely blocked in *npr3 npr4* double mutant plants (Fu et al., 2012). AvrRpt2-induced HR is restored in the *npr3 np4 npr1* triple mutant [9], suggesting that constitutive activation of SA response in *npr3 npr4* mutants is responsible for the suppression of cell death activated by AvrRpt2. This is consistent with reduced ETI-induced cell death in autoimmune mutants with high SA levels.

In conclusion, SA plays dual roles in the regulation of programed cell death in plants. The exact mechanism of how SA regulates cell death is currently still unclear. Analysis of early SA-responsive genes by RNA-sequencing revealed that a large number of positive regulators of defense signaling are strongly up-regulated 1 h after SA treatment (Ding et al., 2018). Induction of these defense regulators may play critical roles in potentiating defense signaling leading to activation of cell death. Meanwhile, many known negative regulators of plant immunity are also rapidly induced after SA treatment. Induction of such negative immune regulators could lead to negative feedback regulation of defense responses and cell death, which is critical in controlling the magnitude of cell death and preventing the spread of cell death beyond the infection site. The key regulatory components downstream of the SA receptors that are involved in SA-mediated inhibition of ETI-induced cell death remain to be determined in the future.

## Author Contributions

YZ designed the experiments. AR performed the experiments. All authors wrote the manuscript.

## Conflict of Interest Statement

The authors declare that the research was conducted in the absence of any commercial or financial relationships that could be construed as a potential conflict of interest.
